# Diagnosis of primary vitreoretinal lymphoma masquerading infectious retinitis by retinal biopsy

**DOI:** 10.1186/s12348-024-00389-y

**Published:** 2024-02-07

**Authors:** Nam V. Nguyen, Farid Khan, Andrew Cannon, Ye Huang, Lucas Kim, Rena Xu, Pukhraj Rishi, Christopher D. Conrady, Timothy C. Greiner, Ana Yuil-Valdes, Steven Yeh

**Affiliations:** 1 College of Medicine, University of Nebraska Medical Center, Omaha, NE USA; 2 Department of Ophthalmology, Stanley M. Truhlsen Eye Institute, University of Nebraska Medical Center, Omaha, NE USA; 3 Department of Laboratory Medicine and Pathology, Mayo Clinic, Rochester, MN USA; 4 Mercer University School of Medicine, Macon, GA USA; 5 Department of Pathology and Microbiology, University of Nebraska Medical Center, Omaha, NE USA

**Keywords:** Retinal biopsy, Vitreoretinal lymphoma, Infectious retinitis

## Abstract

**Purpose:**

To report a case of primary vitreoretinal lymphoma masquerading as infectious retinitis that was diagnosed via a retinal biopsy.

**Observations:**

A 72-year-old female patient was referred to our ophthalmology clinic for evaluation of retinitis and vasculitis in the right eye (OD). On examination, best-corrected visual acuities (BCVAs) were hand motions OD and 20/20 in the left eye (OS). Fundus examination revealed optic disc edema and diffuse retinal whitening superior to the superotemporal arcade OD. Given the high suspicion of infectious retinitis, the patient was treated with intravitreal foscarnet, systemic acyclovir, and oral prednisone and underwent a comprehensive uveitis workup, which was unremarkable for viral and autoimmune entities. Given the patient’s history of diffuse large B cell lymphoma with cutaneous involvement, vitreoretinal lymphoma was suspected, prompting pars plana vitrectomy with a retinal biopsy. Biopsy and immunohistochemistry results were consistent with B-cell lymphoma, and the patient was treated with high-dose methotrexate and rituximab. At 5-month follow-up, BCVAs were hand motions OD and 20/30 OS, and fundus examination demonstrated disc edema with resolution of retinal whitening OD. She responded well to the treatment with regression of vitreoretinal lymphoma on examination and is being monitored closely for lymphoma recurrence.

**Conclusions and importance:**

Although uncommon, patients with vitreoretinal lymphoma may masquerade as infectious retinitis, and vitreoretinal lymphoma should be suspected when refractory to antiviral therapy and in the setting of a negative workup for viral etiologies. Vitrectomy with retinal biopsy may be considered to aid the diagnosis of vitreoretinal lymphoma although careful consideration of the risks and benefits is warranted.

**Supplementary Information:**

The online version contains supplementary material available at 10.1186/s12348-024-00389-y.

## Introduction

Intraocular lymphoma is a rare malignancy accounting for fewer than 1% of all intraocular tumors [1]. Depending on the extent and anatomic location of the posterior involvement, intraocular lymphoma is further classified as vitreoretinal lymphoma or choroidal lymphoma [2, 3]. The diagnosis of intraocular lymphoma has been challenging to clinicians since it can masquerade as a uveitis syndrome or infrequently as infectious retinitis [4–8]. While the diagnosis of vitreoretinal lymphoma may be made via histologic and immunochemical confirmation via a vitreous sample obtained through diagnostic vitretomy [9], the low sensitivity may confound the diagnosis leading to delays in the diagnosis and treatment [10–12].

Vitreoretinal lymphoma can infrequently masquerade as infectious retinitis making management and diagnosis in these patients very challenging [4–7]. When suspicion remains high even with a negative vitreous biopsy, retinal biopsy can be undertaken to diagnose the disease. However, the risks of retinal detachment, hemorrhage, and post-surgical inflammation warrant careful consideration prior to proceeding with surgery. Herein, we describe a case of primary vitreoretinal lymphoma that masqueraded as viral retinitis and was diagnosed via retinal biopsy.

## Case report

A 72-year-old female patient was referred to our ophthalmology clinic for evaluation of retinitis and vasculitis in the right eye (OD).

The patient initially developed blurred vision OD one month prior to presenting to an outside ophthalmology clinic. She was diagnosed with anterior uveitis OD, and topical prednisolone acetate was subsequently initiated. However, her vision continued to deteriorate, and retinal whitening was noted three weeks later. Uveitis work-up, including *Treponema pallidum* IgG, antinuclear antibodies (ANA), and Toxoplasmosis serology, was all negative, and the patient was promptly referred to our ophthalmology clinic for further evaluation.

**Fig. 1:**
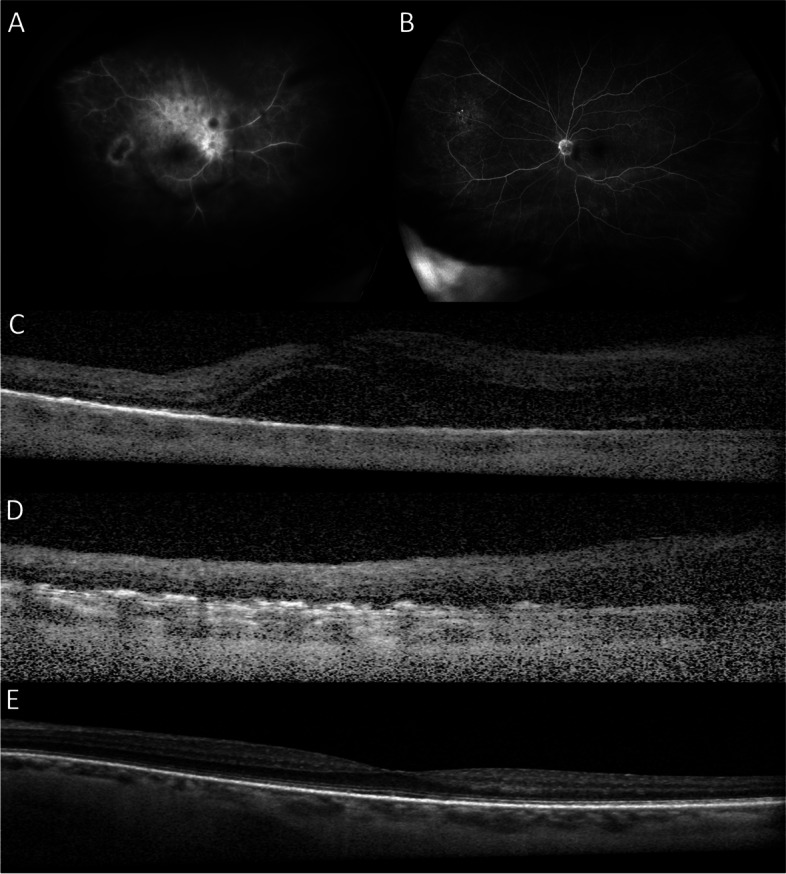
Fluorescein angiography revealed diffuse retinal vascular leakage and hyperfluorescent staining of the corresponding retinal whitening OD (**A**). Optical coherence tomography demonstrated intraretinal fluid and subretinal fluid along with the optic disc edema OD (**C**). The B-scan through the retinal whitening area demonstrated significant retinal thickening with subretinal deposits OD (**D**). Fluorescein angiography of the left eye demonstrated mild hyperfluorescent staining in the periphery, and OCT of the left eye was normal (Fig. 1**B**, **E**)

Detailed past medical history revealed hypertension, hyperlipidemia, hearing loss, chronic cystitis, and diffuse large B cell lymphoma (DLBCL) with cutaneous involvement. She had previously received chemotherapy from February to July 2021 for her DLBCL and had been in remission since. On examination, best-corrected visual acuities (BCVAs) were hand motions OD and 20/20 in the left eye (OS). Slit-lamp examination revealed diffuse fine keratic precipitates, 1 + anterior chamber cells, and 3 + vitreous cells OD. Fundus examination of the right eye was limited due to the vitreous opacities, but optic disc edema and diffuse retinal whitening superior to the superotemporal arcade were noted. Fluorescein angiography of the right eye revealed diffuse retinal vascular leakage and hyperfluorescent staining of the corresponding retinal whitening (Fig. 1A). Optical coherence tomography (OCT) demonstrated intraretinal fluid and subretinal fluid along with the optic disc edema OD (Fig. 1C). The B-scan through the retinal whitening area demonstrated significant retinal thickening with subretinal deposits (Fig. 1D). Fluorescein angiography of the left eye demonstrated mild hyperfluorescent staining in the periphery, and OCT of the left eye was normal (Fig. 1B, E). Given the high suspicion of infectious retinitis, the patient was treated with intravitreal foscarnet, valacyclovir, and oral prednisone and underwent a comprehensive uveitis workup. Magnetic resonance imaging of the brain and orbits demonstrated microvascular changes, and echocardiogram and carotid ultrasound were unremarkable. Furthermore, the patient underwent a lumbar puncture with cerebrospinal fluid analysis, which was suspicious for a B-cell lymphoproliferative disorder.

**Fig. 2:**
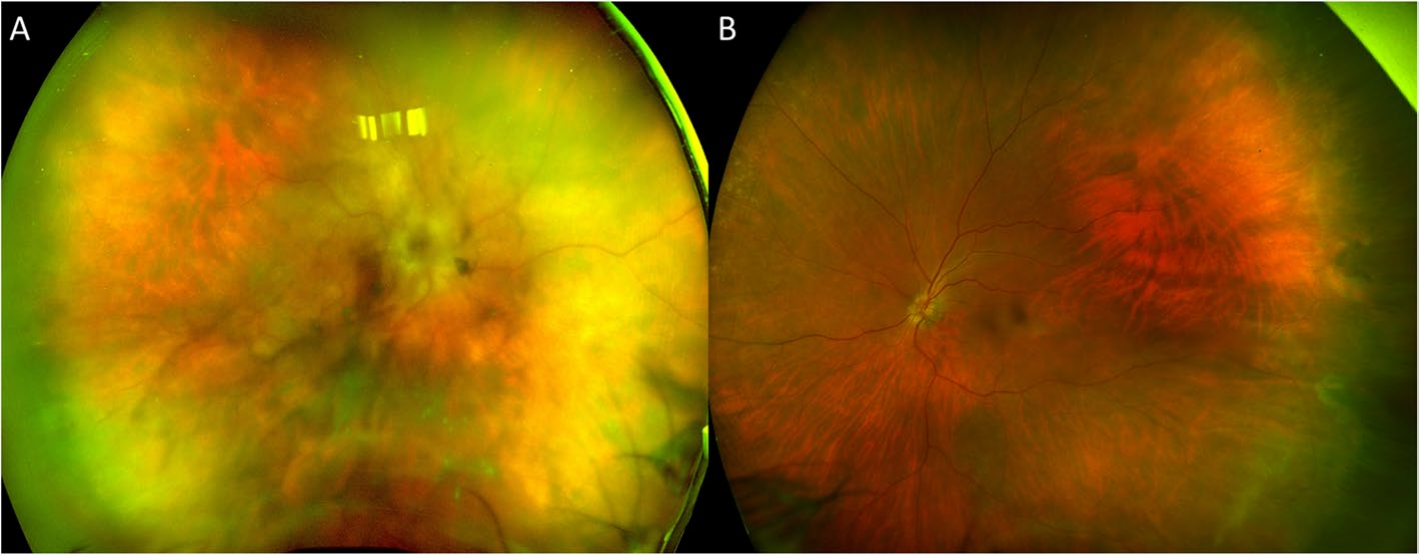
Ultrawide-field color fundus photo of the right eye revealed severe vitritis, optic disc edema with the blurring of margins, whitening around the peripapillary region, and diffuse whitening superior to the superotemporal arcade (**A**). Ultrawide-field color fundus photo of the left eye demonstrated mild vitritis along with some infiltrates in the periphery (**B**)

The patient returned for a follow-up one week later, and her vision remained unchanged. Uveitis work-up was unremarkable including antineutrophilic cytoplasmic antibody (ANCA), antinuclear antibody (ANA), anti-cyclic citrullinated peptide (anti-CCP), rheumatoid factor (RF), Quantiferon-TB, serum Toxoplasmosis IgG and IgM, *Treponema pallidum* antibody, *Bartonella henselae* antibody, angiotensin-converting enzyme (ACE), and Lysozyme. Anterior chamber paracentesis was negative for varicella zoster virus (VZV), cytomegalovirus (CMV), herpes simplex virus (HSV)-1 and HSV-2. On examination, BCVAs were hand motion OD and 20/20 OS. Slit-lamp examination revealed diffuse fine keratic precipitates, trace AC cells, and 2 + vitreous cells OD. Fundus examination of the right eye revealed optic disc edema, whitening around the peripapillary region, and diffuse whitening superior to the superotemporal arcade (Fig. 2A). Examination of the left eye demonstrated mild vitritis along with some infiltrates in the periphery (Fig. 2B). Given the negative PCR testing and history of B-cell lymphoma, vitreoretinal lymphoma was suspected, and the patient was scheduled to undergo vitrectomy with vitreous and retinal biopsy.

A 25-gauge pars plana vitrectomy was performed to obtain a vitreous specimen. The specimen was sent for cytopathologic analysis, flow cytometry, and culture. Following a complete vitrectomy, the retina was visualized, and a zone of retinal whitening was noted. Given the high suspicion for a neoplastic process, decision was made to proceed with a retinal biopsy at the border of abnormal and more normal-appearing retina. Internal cautery was used to demarcate the boundaries of the retinal biopsy. Subsequently, a Finesse loop (Alcon, Ft. Worth, Texas) and intraocular forceps were used to dissect the retina and the underlying pigment epithelium from the choroid. A broad chorioretinal adhesion at the site of the biopsy was noted. The specimen was removed from the eye via a pars plana sclerotomy and was sent for cytopathologic analysis. The donor site was surrounded by three rows of endolaser retinopexy. Fluid-air exchange and 12% perfluoropropane (C3F8) gas exchange were performed (Video, Supplemental Material).

**Fig. 3:**
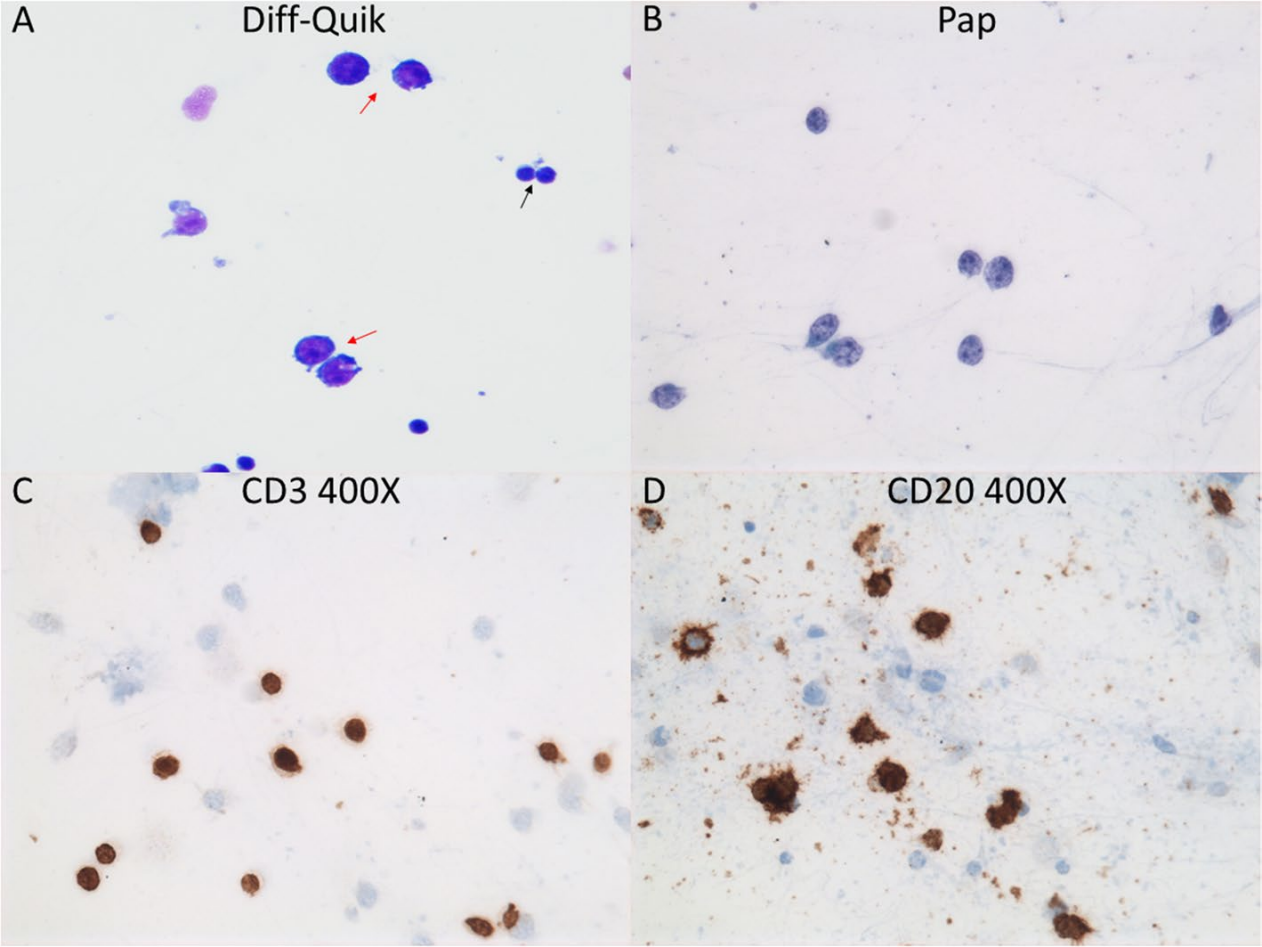
The cytospin preparation showed large, atypical lymphocytes (red arrows) with high nuclear/cytoplasmic ratio and irregular nuclear membranes. A few small benign lymphocytes are present (black arrow) in Diff Quick and Papanicolaou stains (**A**, **B**). Immunocytochemistry for CD20 and CD3 showed robust staining of atypical cells with CD20 (**C**) while small background lymphocytes were reactive with CD3 (**D**)

**Fig. 4:**
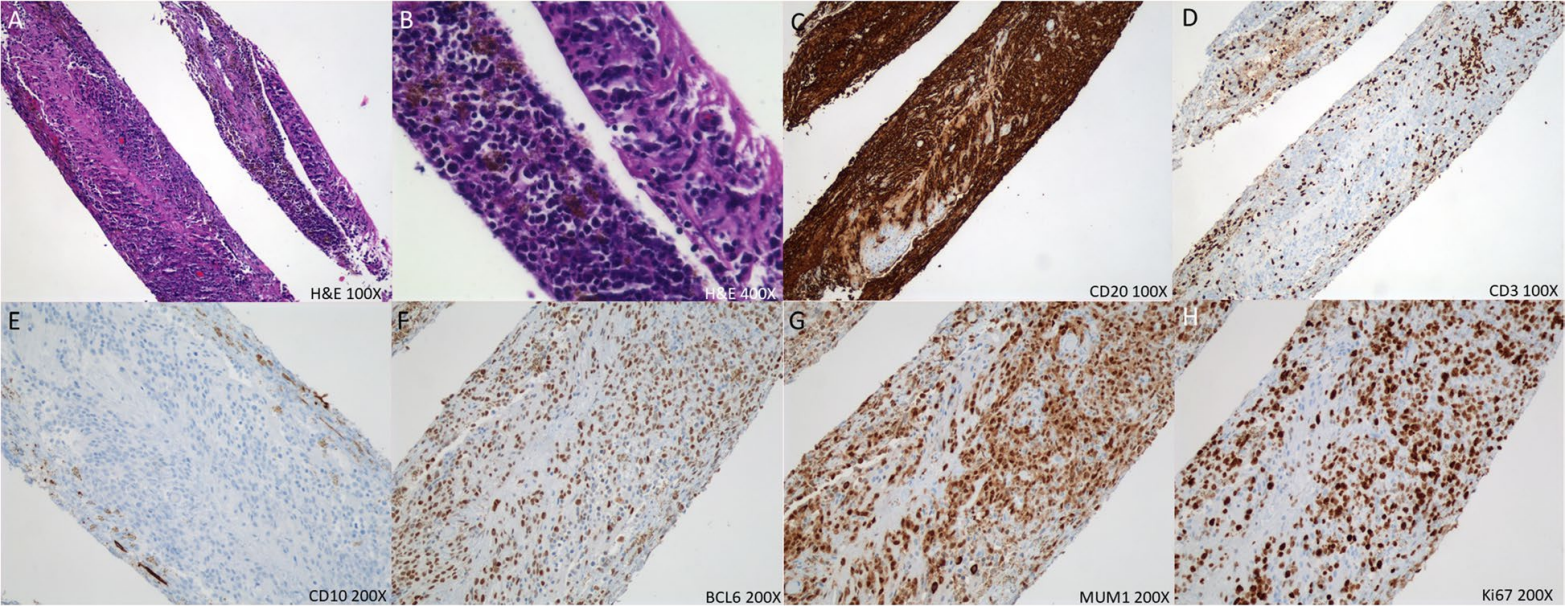
Histologic examination of retinal biopsies demonstrated near complete effacement of retinal architecture by large, atypical lymphocytes (**A**, **B**). These atypical cells were CD20-positive (**C**) while CD3-stained cells were reactive with small, background T-lymphocytes (**D**). The atypical cells were CD10-negative (**E**), 60%-70% of cells stain for BCL6 (**F**) and greater than 80% of cells were reactive with MUM1 (3G). Ki67-stained cells were markedly elevated (**H**). (BCL-6: B-cell lymphoma 6; MUM1: multiple myeloma oncogene-1; Ki67: Antigen Kiel 67)

**Fig. 5:**
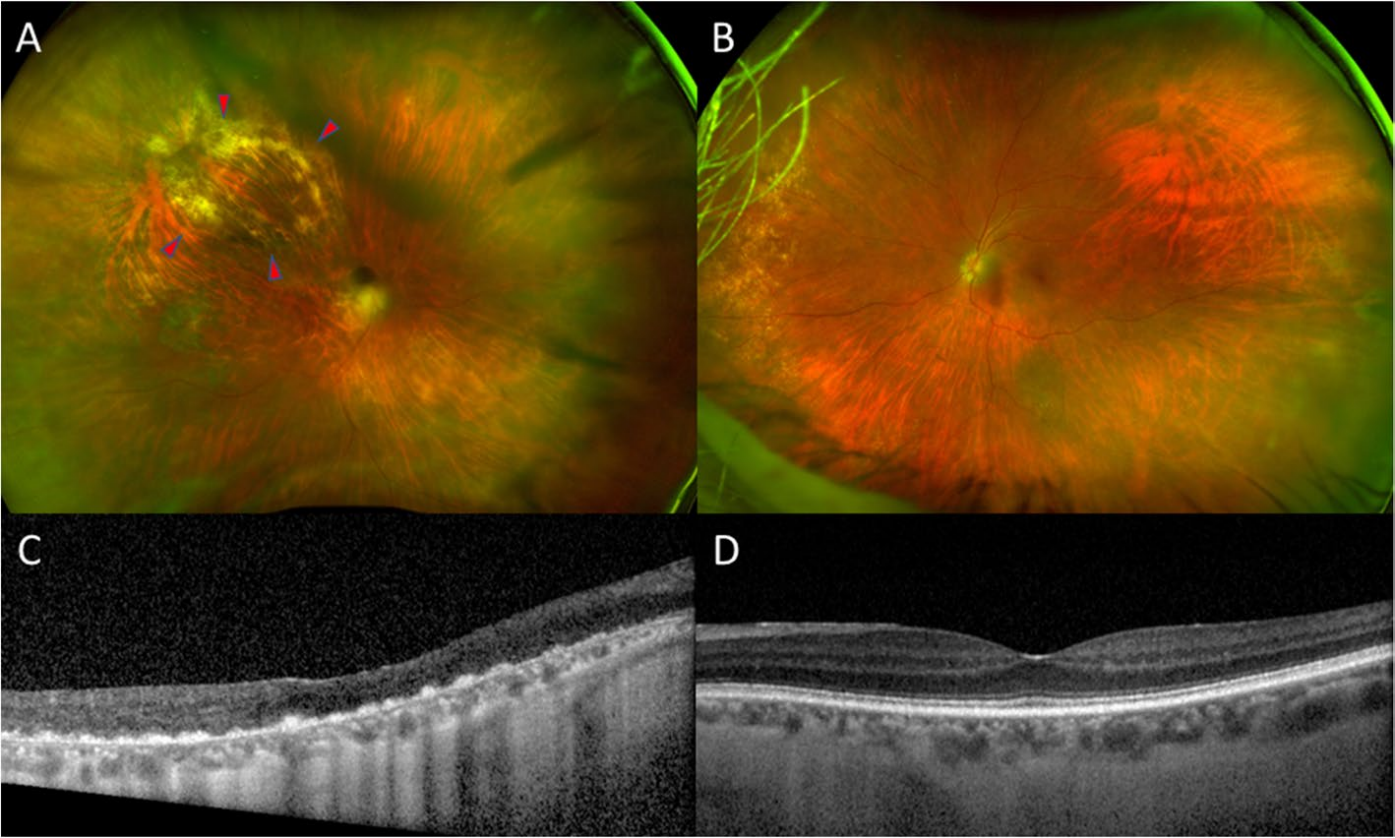
Ultrawide-field color fundus photo of the right eye demonstrated disc edema with blurry margins and resolved retinal whitening, and arrowheads indicated the area of laser where retinal biopsy was taken (**A**). OCT of the right eye revealed loss of outer retinal tissue and resolution of macular edema (**C**). Examination of the left eye remained unchanged (**B**, **D**)

The flow cytometric analysis identified a population of mature large B-cells (77% of isolated cells) with increased side light scatter expressing HLA-DR, CD19, CD38 and moderate-density kappa light chains. Cytology of vitreous fluid demonstrated atypical lymphoid cells with a high nuclear/cytoplasmic ratio, irregular nuclear membranes and multiple nucleoli (Fig. 3A, B). The large lymphoid cells were positive for CD20 (Fig. 3C), while CD3-stained cells were small, reactive T-cells (Fig. 3D). In the retinal biopsy, there was near complete effacement of retinal architecture by large, atypical CD20 + lymphocytes (Fig. 4A, B). Cells of interest were kappa restricted by flow cytometry (Kappa + : 91.55%), and CD10-negative, 60% BCL6 + and > 80% MUM1 + by IHC indicative of a non-germinal center (activated B-cell) phenotype (Fig. 4E, F, G) [13]. Consistent with this diagnosis, Ki67 staining was markedly elevated at 60% positive cells, and the Ki67 proliferative index was 80% (Fig. 4H). The findings were consistent with diffuse large B-cell lymphoma. The patient was diagnosed with vitreoretinal lymphoma and treated with 6 intravenous infusions of high-dose methotrexate (3 g/m^2^) and 6 intravenous infusions of rituximab (375 mg/m^2^) every two weeks. At 5-month follow-up, BCVAs were hand motions OD and 20/30 OS, and slit-lamp examination revealed resolved keratic precipitates, and trace AC cells OD. Fundus examination demonstrated disc edema with blurry margins and resolved retinal whitening OD (Fig. 5A). OCT of the right eye revealed loss of outer retinal tissue and resolution of macular edema (Fig. 5C). Examination of the left eye remained unchanged (Fig. 5B, D). She responded well to the treatment with regression of vitreoretinal lymphoma on examination, and she was monitored closely for lymphoma recurrence. Unfortunately, she developed recurrence 5 months later, which subsequently prompted intravitreal methotrexate (400 mcg/0.1 mL) monthly for 3 months and oral maintenance chemotherapy with ibrutinib.

## Discussion

In this report, we have described a case of primary vitreoretinal lymphoma masquerading as an infectious retinitis that was diagnosed via a retinal biopsy. Vitreoretinal lymphoma can often mimic a uveitis syndrome that makes the clinical diagnosis of vitreoretinal lymphoma difficult for the clinicians [4–8]. Secondary vitreoretinal lymphoma may also mimic an infectious retinitis, that may be diagnosed by vitrectomy or a retinal biopsy [4, 14].

Our patient presented with an area of retinal whitening on fundus examination, and fluorescein angiography demonstrated hyperfluorescent staining of the area. The B-scan through the retinal whitening area also showed subretinal deposits. Given these multimodal imaging findings, infectious retinitis was suspected; however, anterior chamber paracentesis was negative for infectious etiologies and clinical symptoms did not improve upon initiation of antiviral medication and oral corticosteroids. Given the patient’s medical history of DLBCL with cutaneous involvement, our suspicion of vitreoretinal lymphoma was high and prompted urgent vitrectomy with vitreous and retinal biopsy. Interestingly, the large B-cell lymphoma from the previous year was CD10-positive meanwhile the current lymphoma was CD10-negative, and phenotype indicated non-germinal center subtype (activated B-cell subtype). This finding suggests that the current disease was a new-onset lymphoma as opposed to a recurrence of the previous DLBCL.

Vitreous biopsy with cytology is frequently utilized to establish a diagnosis of intraocular lymphoma [9]; however, a significant portion of the patients may have false negative results further delaying the diagnosis and treatment [10–12]. Moreover, the fragility and paucity of the vitreous lymphoma cells present another challenge in preserving the tissue for cytologic analysis [15]. Other studies have proposed the use of IL-10:IL-6 ratio as a laboratory tool for the diagnosis of PVRL, but the results have been inconsistent throughout multiple studies [16–18]. In a study by *Cassoux *et al., 51 vitrectomy specimens from patients with suspicion of vitreoretinal lymphoma were included in the study, and an IL-10 cut-off value of 400 pg/ml correlated with 80% sensitivity and 99% specificity [17]. In another study by *Pochat-Cotilloux *et al., an IL-10 cut-off value of 65 pg/ml and 30 pg/ml in the vitreous and aqueous humors was associated with sensitivity of 93% and 78%, respectively, and specificity of 100% and 97%, respectively [19]. An IL-10/IL-6 ratio higher than 1 was shown to be associated with sensitivity of 93% and specificity of 100% [19]. Another scoring system termed – the Interleukin Score for intraOcular Lymphoma Diagnosis (ISOLD) – was also proven to be promising in predicting the likelihood of intraocular lymphoma based on IL-10 and IL-6 value [20]. Several studies have proposed the use of polymerase chain reaction (PCR) to detect MYD88 L265P mutation in aqueous and vitreous fluids to diagnose and monitor vitreoretinal lymphoma [21–25]. In a study by *Hiemcke-Jiwa *et al., MYD88 L265P was detected in 74% of patients with cytology/biopsy-proven vitreoretinal lymphoma, and it was not detected in patients with uveitis [23]. Moreover, the detection of MYD88 L265P showed a sensitivity of 75% and a positive predictive value of 100% in the vitreous sample, and a sensitivity of 67% and a positive predictive value of 100% in the aqueous sample [23]. Interestingly, MyD88 testing was shown to have the lowest coefficient of variation (CV) among different diagnostic tests for vitreoretinal lymphoma [26]. The coefficient of variation for MyD88 was 0.15 meanwhile CV of other diagnostic tests including cytology, PCR IgH rearrangement, flow cytometry and IL10/IL6 ratio ranged from 0.23–0.44 [26]. This finding highlights the potential of MyD88 in improving the diagnosis of vitreoretinal lymphoma.

Previous case reports have used retinal biopsy in diagnosing primary vitreoretinal lymphoma when suspicion remains high after a negative vitreous biopsy [4, 6, 27, 28]. In a retrospective study by *Mastropasqua *et al., 29 patients with suspicion of intraocular lymphoma were included in the study, and they found that chorioretinal biopsy was able to make a specific histologic diagnosis in 17 cases while excluding malignancy in 9 cases [29]. Only 3 patients had an inconclusive biopsy in the study. These findings suggest that even though it is not recommended as a first-line diagnostic tool if a diagnosis can be established by vitrectomy alone, a retinal biopsy may be considered in certain situations, particularly in patients with a retinitis that does not respond to anti-infective therapies or patients with a high suspicion of vitreoretinal lymphoma with negative vitreous biopsy. Given the potential for a false negative vitreous biopsy and our patient’s severe visual impairment with rapid progression, the decision was made to proceed with retinal biopsy to prevent further delay of diagnosis and treatment for the patient. Nonetheless, risks including retinal detachment, hemorrhage, and postoperative inflammation are important considerations prior to undertaking this invasive procedure. Extensive counseling of the risks of a retinal biopsy were discussed with the patient prior to proceeding in this case.

## Conclusions

In conclusion, we have described a case of primary vitreoretinal lymphoma mimicking infectious retinitis that was ultimately diagnosed via a retinal biopsy. Although uncommon, patients with vitreoretinal lymphoma can masquerade as infectious retinitis, and vitreoretinal lymphoma should be suspected when refractory to antiviral therapy. Although it is not recommended as a first-line, retinal biopsy can be considered in these diagnostic challenging situations to aid the diagnosis of vitreoretinal lymphoma.

### Supplementary Information

**Additional file 1.**
